# Use of mobile technology to identify behavioral mechanisms linked to mental health outcomes in Kenya: protocol for development and validation of a predictive model

**DOI:** 10.1186/s13104-023-06498-6

**Published:** 2023-09-21

**Authors:** Willie Njoroge, Rachel Maina, Elena Frank, Lukoye Atwoli, Zhenke Wu, Anthony K Ngugi, Srijan Sen, JianLi Wang, Stephen Wong, Jessica A Baker, Eileen M Weinheimer-Haus, Linda Khakali, Andrew Aballa, James Orwa, Moses K Nyongesa, Jasmit Shah, Akbar K Waljee, Amina Abubakar, Zul Merali

**Affiliations:** 1 Brain and Mind Institute, Aga Khan University, Nairobi, Kenya; 2 Department of Psychology, Southwest University, Chongqing, China; 3 Department of Methodology and Statistics, Tilburg University, Tilburg, The Netherlands; 4 Michigan Neuroscience Institute, University of Michigan, Michigan, USA; 5 Department of Medicine, Medical College East Africa, the Aga Khan University, Nairobi, Kenya; 6 Department of Biostatistics, University of Michigan, Ann Arbor, MI USA; 7 Department of Population Health, Aga Khan University, Nairobi, Kenya; 8 Department of Community Health and Epidemiology, Dalhousie University, Halifax, Canada; 9 Computing and Data Innovation Office, Aga Khan University, Nairobi, Kenya; 10 Center for Global Health Equity, University of Michigan, Ann Arbor, MI USA; 11 Department of Learning Health Sciences, University of Michigan Medical School, Ann Arbor, MI USA; 12 Department of Sociology, University of Nairobi, Nairobi, Kenya; 13 Institute for Human Development, Aga Khan University, Nairobi, Kenya; 14 Neurosciences Unit, Kenya Medical Research Institute−Wellcome Trust Research Programme, Kilifi, Kenya

**Keywords:** Mobile Technology, Healthcare Workers, Mental Health, Predictive model, Artificial Intelligence, Machine learning

## Abstract

**Objective:**

This study proposes to identify and validate weighted sensor stream signatures that predict near-term risk of a major depressive episode and future mood among healthcare workers in Kenya.

**Approach:**

The study will deploy a mobile application (app) platform and use novel data science analytic approaches (Artificial Intelligence and Machine Learning) to identifying predictors of mental health disorders among 500 randomly sampled healthcare workers from five healthcare facilities in Nairobi, Kenya.

**Expectation:**

This study will lay the basis for creating agile and scalable systems for rapid diagnostics that could inform precise interventions for mitigating depression and ensure a healthy, resilient healthcare workforce to develop sustainable economic growth in Kenya, East Africa, and ultimately neighboring countries in sub-Saharan Africa. This protocol paper provides an opportunity to share the planned study implementation methods and approaches.

**Conclusion:**

A mobile technology platform that is scalable and can be used to understand and improve mental health outcomes is of critical importance.

## Introduction

According to the World Health Organization (WHO), mental disorders account for the largest burden of illness among all types of health problems [1]. Major depressive disorder is the second leading cause of disability, affecting more than 350 million people worldwide [2]. In low-and-middle-income countries (LMICs), resources are particularly limited for surveillance, diagnosis, and treatment. In Kenya, a report prepared by a national mental health taskforce [3] highlighted the fact that the severe state of neglect of mental health services, infrastructure and research has led to a “national crisis” and implored the mobilization of immediate and effective solutions to address mental health issues. The Taskforce highlighted that research institutions and universities should: (1) collect relevant data to better define the mental illness gaps (in detection and interventions), burden and determinants; (2) focus particular attention on escalating interventions to reduce depression and suicidal acts. We propose a novel mobile infrastructure in Kenya that has previously been deployed in the United States of America (USA), to test its validity and predictive capability in a different cultural setting [4, 5].

**Fig. 1:**
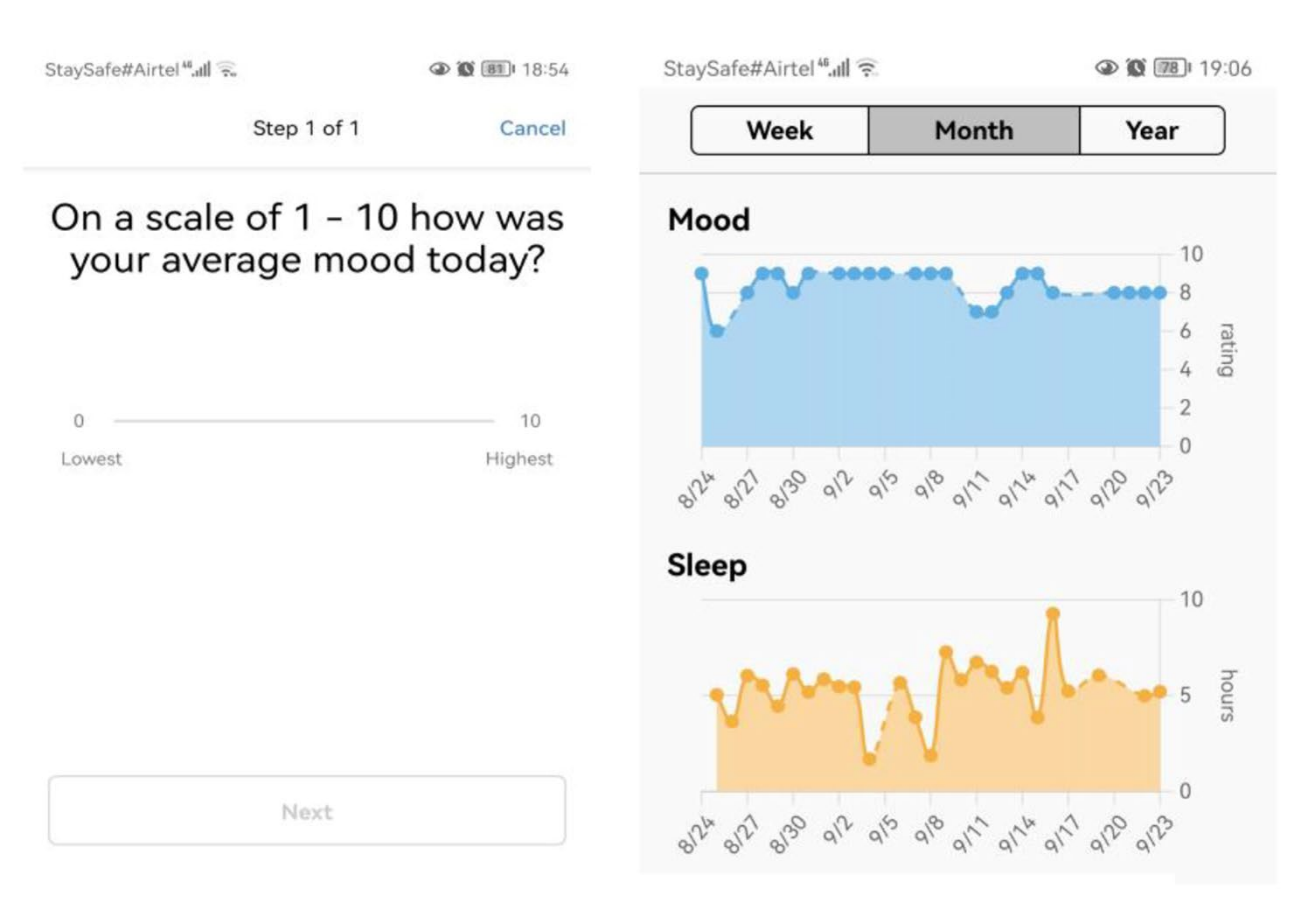
App Dashboard

The proposed study builds up on the Intern Health Study which is a prospective longitudinal cohort study of stress and depression among training physicians in the USA and China [6, 7]. This model allows for the same individuals to be followed, first under normal conditions and then under high-stress conditions [8]. The Intern Health Study employs a unique mobile app platform, the Intern App, specifically designed for healthcare workers to collect and integrate active and passive data on mood, sleep, and activity. Intern Health Study has demonstrated that mobile monitoring can facilitate the prospective, real-time monitoring of continuous, passive measures and effectively predict short-term risk for depressive episodes in a large group of individuals *(See* Tables 1*and* Fig. 1*).* Mobile technology coupled with predictive models can help in triggering early warning systems e.g., signs of depression [8]. We would like to adapt the App and make it contextually relevant and deploy it among healthcare workers within Kenyan urban and semi-urban settings.

The objective of the proposed study is to demonstrate the feasibility of deploying a novel methodology for developing predictive models for mood changes and depression among Kenyan healthcare workers. The specific objectives of the research are:

i) To adapt, validate and refine previously developed Artificial Intelligence/Machine Learning (AI/ML) based prediction models of depression and mood among Kenyan healthcare workers; and.
ii) To collect and evaluate individual active and passive data that may predict depression and mood among Kenyan healthcare workers.

## Main text

### Methods

#### Design

This will be a longitudinal cohort study.

#### Study settings

The setting will be in five urban and semi-urban healthcare facilities in the Kenyan capital city, Nairobi, where healthcare workers from diverse cadres are directly involved with the patient’s care.

##### Facility 1

The Aga Khan University Hospital Nairobi, which is a private facility with a bed capacity of 254 with 14 different specialty departments.

##### Facility 2

Pumwani Hospital, which is a public maternity facility with 396 healthcare staff spread across 13 different cadres (physicians, nurses, nutritionists, and medical students) and 25 different departments [9].

##### Facility 3

Mama Lucy Hospital, which is a public facility with 700 healthcare workers who attend to more than 800 outpatients daily and has an ever-growing bed capacity.

##### Facility 4

Kenyatta National Hospital, which is a public facility which has a bed capacity of 1800 and a staff population of 6000 [10]. These staff give medical care to patients in 50 inpatient wards, 24 theaters, 22 outpatient clinics and 1 accident and emergency center.

##### Facility 5

Kenyatta University Teaching Referral and Research Hospital, which is a public facility which has an over 650 bed capacity and there are around 665 hospital staff [11].

#### Sample size determination

The Cochran formula to estimate a representative sample for proportions is utilized [12]. Using a 95% confidence level, proportion = 0.5 (maximum variability) of +/- 5%, the minimum sample size required will be 462 participants after an attrition rate of 20%. n= (Z^2 p q)/e^2 where *n* is the sample size, *Z* is the statistic corresponding to 95% level of confidence, *P* is expected proportion, and *d* is precision (corresponding to effect size). Thus, we will recruit 500 participants, which will translate to 100 participants from each of the five healthcare facilities.

#### Instruments description

A socio-demographic questionnaire in the App incorporates elements such as age, gender and education background.

The app is the platform utilized to electronically capture all the tools within the participant questionnaire. Utilizing the app in this way enables us to have skip logistics in the questionnaire, making it shorter for the participants. For example, where a participant responds and says that they have never had a COVID-19 vaccination, they will not encounter questions on whether and when they got their first, second and third dose, experience with the vaccine, and type of vaccine. In line with this response process, it will be difficult to identify the total number items in the questionnaire that each participant will attend to. However, we can estimate the time for completion in one sitting as 20–30 min.

Each participant will complete the following standardized instruments/tools:

1. Neuroticism - NEO-Five Factor Inventory [13] is a 60-item measurement that is designed to assess personality in the domains of neuroticism, extraversion, openness, consciousness, and agreeableness. Each item is scored with a 5-point Likert scale ranging from “strongly disagree, disagree, neutral, agree and strongly agree. Internal consistency reliability [13] of the tool has been found to be good α = .86.
2. Positive and Negative Suicide Inventory (PANSI) [14] will measure suicidal ideation. PANSI evaluates both the protective and risk factors associated with suicidal ideation and comprises two dimensions (14 items total): positive ideation (PANSI-PI, 6 items) and negative suicide ideation (PANSI-NSI; 8 items). PANSI-NSI and PANSI-PI examine the frequency of specific negative thoughts (e.g., failure to accomplish something important) or positive thoughts (e.g., excited about doing well at school or work) related to suicidal behavior [14]. Participants will use a Likert scale ranging from 1 (i.e., “none of the time”) to 5 (i.e., “most of the time”) to assess the frequency they experience suicidal ideation. Higher scores indicate more positive or negative suicide ideation, depending on the item’s particular subscale.
3. Patient Health Questionnaire (PHQ-9) [15]. It consists of nine major depression diagnostic items of DSM-V: anhedonia, depressed mood, trouble sleeping, feeling tired, guilt or worthlessness, concentration difficulties, feeling restless and suicidal thoughts. Each item is scored from 0 (not all) to 3 (nearly every day). The final score indicates depressive symptoms: minimal (0–4), mild (5–9), moderate (10–14), moderately severe (15–19) and severe (20–27). PHQ-9 has good reliability and convergent validity [16]. The Swahili PHQ-9 is validated in Kenya and has demonstrated good reliability α = 0.84 [17].
4. Pittsburgh Sleep Quality Index [18] will assess sleep quality in relation to a range of subjective estimations component scores. The scores range 0–21 with higher scores indicating an increased dissatisfaction with sleep and a greater severity of sleep disturbance. Though not validated in Kenya, it has been validated in other African settings such as Ethiopia moderate internal consistency α = 0.59 [19] and Nigeria α = 0.55 [20].
5. Risky Families Questionnaire [21] will be utilized to assess the degree of risk of physical, mental, and emotional distress that participants faced in their homes during childhood and adolescence. Participants will rate the frequency with which certain events or situations occurred in their homes during the ages of 5–15 years, using a 5-Point Likert scale (1 = Not at All, 5 = Very Often). Internal consistency reliability of the tool has been found to be good α = 0.86 [13].
6. Generalized Anxiety Disorder – 7 (GAD-7) [22] will measure anxiety based on seven items which are scored from 0 to 3. The scale score can range from 0 to 21 and cut-off scores for mild, moderate and severe anxiety symptoms are 5, 10 and 15 respectively [22]. GAD-7 has demonstrated good internal consistency and convergent validity in heterogeneous samples [23] and has been used within Kenyan settings [24, 25].
7. MyDataHelps App – Online Survey.

MyDataHelps is an application *(available on iOS, Android, and web)* developed by CareEvolution which provides step-by-step instructions for collecting data from participants via their smartphones [26]. The app acts as a tool for consent, data collection, delivery of study questionnaires, automated notifications, and reminders.

8) Mobile Monitoring.

In addition to completing surveys, the participants will also be asked to wear Fitbit Inspire 2™ (www.fitbit.com) which is a wearable fitness tracker wristband to track daily activity levels, daily mood rating and sleep. The device automatically connects via Bluetooth and transfers data to a mobile platform via a dedicated App. Fitbit Inspire 2™ allows tracking of sleep stages (minutes spent awake, in “light”, “deep”, and “REM” [Rapid Eye Movement] sleep) in addition to sleep/wake states. During set-up, the participants will be prompted to allow MyDataHelps to send them notifications. A notification will be automatically generated on their phone daily and will appear as follows: *“On a scale of 1 to 10 what was your average mood today?”* They will then respond in the App. We will ask them to complete this daily rating for a period of one year. After completing the consenting process and initial survey via the study App, participants will be given Fitbit Inspire 2. They will be asked to sync the fitness tracker with their phone and wear it daily through to the end of their study year for the purpose of collecting objective data on their daily activity. On the App activities page, participants will be able to track their recent activity data (e.g., how many days they entered mood over the past week, last fitness tracker sync) (see *“MyDataHelps App – Dashboard-Activities Screen”*). Healthcare workers will also be able to view graphs of their mood, sleep, and step data at any time on the App dashboard. (**See** Fig. 1)

#### Sampling Procedure

The healthcare workers will be sampled using random sampling technique on the basis that they meet the following inclusion criteria: voluntary consent to participate in the study; enrolled at the sampled facility as a healthcare worker (including residents) and own a smart phone. Exclusion criteria will be: decline consent to participate in study; has a non-smartphone and HCW’s on locum. This sampling technique is preferred due to the large number of healthcare workers who rotate in these sites. Moreover, though there is as high as 117.2% mobile connections in the country, some participants may not have phones with app access and latest operating systems that are compatible with our data collection devices. The country has been, however, reporting an increase in mobile phone access which may support scalability of the study in future as newer phones come with updated features. Potential participants will be randomly selected from a list with staff names and contacts. They will then be contacted via email or smartphone.

#### Data collection procedure

We will administer surveys and collect active and passive mobile data on mood and affect over a 12-month period in a Kenyan cohort of 500 healthcare workers, recruited from five health facilities. The frequency of the data collection will vary i.e., daily, monthly and quarterly. The initial data collection day will be the same as the consenting day. Right after the participants give consent through the App (MyDataHelps), a unique identifier will be generated. All consented healthcare workers will then be encouraged to complete the baseline survey. In addition to demographic and baseline psychological information (Neuroticism using the NEO-FFI and Early Family Environment using the Risk Family Questionnaire), the baseline survey will assess anxiety symptoms using GAD-7, suicidal symptoms using PANSI and depressive symptoms using PHQ-9 [14–18]. Participants will also be asked for permission for us to collect active and passive data through their smartphone. On submission of the survey, the healthcare workers will receive a message directing them where they will pick Fitbit Inspire 2.

**Fig. 2:**
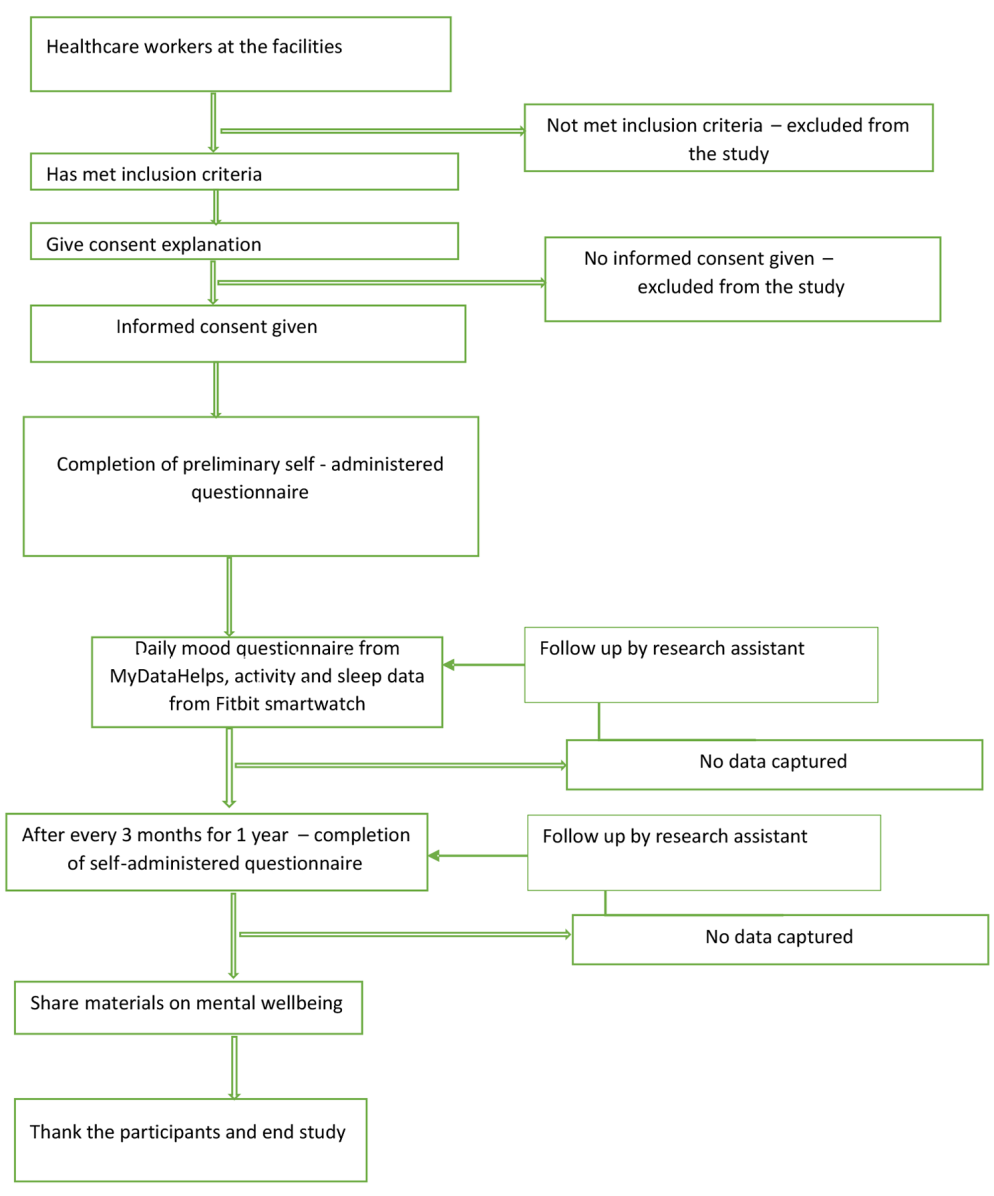
Study flow chart

During the 12-month data collection period, participants will respond daily to a validated one-question measure of mood valence via the mobile App (scale of 1 to 10). At 3-month intervals (quarterly) they will also be assessed via the App using a shorter questionnaire designed to assess: (1) current depressive symptoms measured by the PHQ-9, (2) current suicidal symptoms measured by the PANSI, (3) current anxiety symptoms measured by the GAD-7, (4) non-work life stress, (5) COVID-19 experiences, (6) work hours, and (7) perceived medical errors *(*Table 1*).* If participants do not respond to the surveys and have not indicated that they do not wish to participate in the study, a reminder will be sent after 3 days of no response *(See Fig. 2*). The healthcare workers will be compensated (Kenya Shillings 500.00 or USD $4.06 equivalent) for each quarterly survey. Compensation has been shown to reduce attrition rate [27] which may be felt in this study due to lack of resources to buy internet access tokens. In a country where internet access is intermittent and not always freely available, the compensation will aid in having ready internet access during data upload.

**Table 1:** Summary of longitudinal data to be collected in the study

Source (Device/App)	Measure	Active or Passive	Description	Timing
MyDataHelps	Study Consent	Active	IRB Consent on mobile form	Enrollment
MyDataHelps	Demographics	Active	Survey	
MyDataHelps	Neuroticism	Active	NEO-Five Factor Inventory	Enrollment
MyDataHelps	Early Family Environment	Active	Risk Family Questionnaire	Enrollment
MyDataHelps	Depression	Active	Patient Health Questionnaire (PHQ-9)	Enrollment
MyDataHelps	Anxiety	Active	Generalized Anxiety Disorder-7 (GAD-7)	Enrollment
MyDataHelps	Suicidal symptoms	Active	Positive and Negative Suicidality Inventory (PANSI)	Enrollment
MyDataHelps	Stressful life events	Active	Survey	Enrollment
MyDataHelps	COVID-19 experiences	Active	Survey	Enrollment
MyDataHelps	Work hours	Active	Survey	Enrollment
MyDataHelps	Perceived medical errors	Active	Survey	Enrollment
MyDataHelps	Daily mood	Active	Subject contacted via push notification	Daily
Fitbit Inspire 2	Heart rate	Passive	Heart rate variability	Continuous
Fitbit Inspire 2	Physical activity	Passive	Hourly steps; activity level (light, moderate, heavy)	Continuous
Fitbit Inspire 2	Sleep	Passive	Bedtime; wake time; total sleep time; restless time; estimated REM, deep, and non-REM sleep	Continuous

IRB = Institutional Review Board; REM = Rapid Eye Movement; non-REM = None-Rapid Eye Movement; COVID-19 = Corona Virus Disease 2019

#### Data analysis and presentation

Data will be collected, stored in the cloud database, and checked for completeness daily. Whenever we come across missing data, a reminder will be sent to the respondents after three consecutive days of missed entries, urging them to complete the data entry process. When they opt out of the study, reminders will no longer be sent. Once the study is over, the data will be stored in accordance with guidelines from Kenya’s Data Protection Act 2019 [28], National Institute of Mental Health and Aga Khan University Hospital. For aim 2, we will adapt and test models using mobile data in the domains that are most promising as predictors (sleep behaviors, mood, anxiety) of depression and future mood from the Intern Health Study. This will be used to determine if the predictors identified in the Intern Health Study dataset suggest similar correlates in our Kenyan sample, which would be essential in adapting, developing, and validating individualized risk prediction models for depression and mood. We will identify data driven behavioral phenotypes, derived from mobile data elements, which predict short-term risk for mood changes and depressive episodes.

*Modeling approach*. The framework for this model was built in 1999 [29, 30], and has been constantly updated.

*Circadian phase, sleep drive and performance.* We will use our modeling framework to estimate circadian phase and sleep drive in individual subjects. We will compare the Fitbit estimates of individual’s asleep and awake durations, with our predicted sleep drive. The predicted relationship between these variables by our mathematical models will be compared with these datasets, aggregated over the population to refine parameters for the mathematical model, which will represent the behavior of a typical individual. These estimates of circadian and sleep drive have the potential to inform shift work.

*Behavioral phenotyping for Mood Prediction.* We will make inferences about which behavioral phenotypes are most predictive and evaluate their time-delayed impact upon mood at different time lags. Granger causality [31] is a quantitative framework for assessing the relationship between time series, and has been widely used in econometrics, neuroscience, and genomics to study the temporal causal relations among multiple economic events [31, 32] directional interactions of neurobiological signals [33–35] and gene time-course expression levels [36–38], respectively. Given two time series $\text{X}$ and $\text{Y}$, the temporal structure of the data is used to assess whether the past values of $\text{X}$, are predictive of future values of $\text{Y}$, beyond what the past of $\text{Y}$ can predict alone; if so, $\text{X}$ is said to *Granger cause*$\text{Y}$. Consider the two regression models: (a) ${\text{Y}}_{\text{T}}=\text{A}{\text{Y}}_{1:\text{T}-1}+\text{B}{\text{X}}_{1:\text{T}-1}+{\text{?}}_{\text{t}}$ and (b) ${\text{Y}}_{\text{T}}=\text{A}{\text{Y}}_{1:\text{T}-1}+{\text{?}}_{\text{t}}$, where A is a vector with *j*-th element is the lag-*j* effect upon ${\text{Y}}_{\text{T}}$ and B is a vector with *j*-th element representing the effect of *j*-th lag of X upon Y. Then X is said to be Granger-causal for Y only if model (a) results in significant improvement over model (b). We will use the multivariate extension of the notion of Granger causality between two variables to *p* variables, referred to as graphical Granger models (GGM) [39]. GGM uncovers a sparse set of Granger causal relationships amongst the individual univariate time series, for example, to establish the temporal relationship between the daily sleep hours and mood scores. In the GGM, on a given day, a behavioral phenotype is said to be Granger causal for another if the corresponding coefficient for this phenotype and day is significant.

*Feature and temporal order selection*. We will estimate the strength of association among the mobile phenotypes and their respective associations with the daily mood scores. For example, we will construct the sequence of sleep hours averaged over the past 1, 2 or 7 days and study their respective association with self-reported mood scores. These estimates will directly address two questions: (1) “Which phenotypes and their temporal features predict the daily mood scores?”, and (2) “If a phenotype is predictive of daily mood scores, at what time lags do we observe the strongest predictive strength?” We address these questions via Lasso-type estimates in the context of Granger causality to estimate the effects of variables on each other in a sparse graphical model framework [37]. Important questions including the direction (positive/negative) and the strength of the effect of, say sleep hours on mood scores, can then be answered. Such methods have been known to reduce false positive and false negative rates [37] in inferring the relations among mobile phenotypes and between the mobile phenotypes and the daily mood scores.

*Nonlinearity extensions*. We will also likely observe nonlinear relationships between the mobile phenotypes and the daily mood scores. We will use kernel-based regression methods to explicitly relax the linear dependence of the outcome upon predictors [40].

*Latent variable model for integrating mood scores and PHQ-9 responses*. In addition to the daily mood scores, we will also use PHQ-9 scores to assess the study participants’ depressive symptoms. We will use dynamic latent variable models [41] to address the different frequencies at which daily mood scores and PHQ-9 data are collected. We will introduce a daily latent variable that represents the true mood for every subject. The true daily mood will then be informed by both the self-reported mood score and the proximal PHQ-9 survey responses. This approach has been applied to the study of complex disease etiology where multiple measurements with different errors and frequencies are integrated to provide extra statistical precision [39, 42]. The integrated analysis has the advantage of increasing the power of identifying important mobile phenotypes that are predictive of the true mood represented by the latent variable.

### Study limitations

*Selection and Missing Data Bias.* As with in any longitudinal study, missing data may be a problem. We will perform a secondary analysis after accounting for missing data to ensure the absence of missing data biases and to maximize power [43].

*Self-Reported Depressive Symptoms.* We cannot diagnose major depressive disorders (MDD) using self-reported assessments. However, our self-reporting assessment has several advantages. For instance, the PHQ-9 instrument that we will utilize to measure depressive symptoms has a sensitivity of 88% and a specificity of 88% for a MDD diagnosis with a cut-off of 10 points or higher [15, 44]. Further, there is evidence that in the population of young healthcare workers, maintaining anonymity is of paramount importance to the accurate assessment of sensitive personal information such as depressive symptoms, suggesting that self-reporting may be the most accurate ascertainment method in this population [45].

*Accuracy of Mobile Data.* The smartphone and wearable devices utilized in this study rely on emerging technology and do not measure parameters of sleep and activity as accurately as gold-standard laboratory tests [46, 47]. While these mobile tools can be used at a scale and in settings that traditional assessment cannot, preliminary evidence suggests that mobile tools produce meaningful and important data [48], we will rigorously monitor their accuracy and upgrade to better technology as it becomes available.

Generalizability: The study healthcare workers are domiciled in hospitals within Nairobi which is a setting characterized by urban and semi-urban dwellings. The results may not depict the mental health status of healthcare workers in rural settings, hence limiting generalizability of our findings. We have however made efforts to enlist participation from the different hospital levels to include public and private hospitals and diverse cadres in ensuring generalizability in respect to hospital infrastructure and staffing and potential scalability in similar health facilities and staffing in urban and semi-urban settings.

#### Response Bias

We anticipate Hawthorne effects when participants change patterns of behavior due to having devices that they can utilize in monitoring and improving their own wellbeing. There is also a possibility of participants choosing not to wear their Fitbits at certain times due to fatigue or discomfort during sleep. Some of these response behaviors will be difficult to mitigate. However, we have integrated adherence schedules that will help us in following up participants with missing data points for more than a week.

## Data Availability

There is no data for sharing now as no data sets have been generated or analyzed.

## List of abbreviations

UM: University of Michigan
USA: United States of America
MDD: Major Depressive Disorders IHS:Intern Health Study
PANSI: Positive and Negative Suicidality Inventory
GAD 7: Generalized Anxiety Disorder version 7
PHQ-9: Patient Health Questionnaire version 9
AI/ML: Artificial Intelligence and Machine Learning
KNH/UON-ERC: Kenyatta National Hospital and University of Nairobi Ethics Review Board
NACOSTI: National Commission for Science, Technology and Innovation
FIC: Fogarty International Center
AKU: Aga Khan University
NIBIB: National Institute of Biomedical Imaging and Bioengineering
NIMH: National Institute of Mental Health
OD: Office of The Director, National Institutes of Health
LMICs: Low-and-Middle-Income Countries
