# Correction: Use of mobile technology to identify behavioral mechanisms linked to mental health outcomes in Kenya: protocol for development and validation of a predictive model

**DOI:** 10.1186/s13104-024-06731-w

**Published:** 2024-03-12

**Authors:** Willie Njoroge, Rachel Maina, Elena Frank, Lukoye Atwoli, Zhenke Wu, Anthony K Ngugi, Srijan Sen, JianLi Wang, Stephen Wong, Jessica A Baker, Eileen M Weinheimer-Haus, Linda Khakali, Andrew Aballa, James Orwa, Moses K Nyongesa, Jasmit Shah, Akbar K Waljee, Amina Abubakar, Zul Merali

**Affiliations:** 1 Brain and Mind Institute, Aga Khan University, Nairobi, Kenya; 2 Department of Psychology, Southwest University, Chongqing, China; 3 Department of Methodology and Statistics, Tilburg University, Tilburg, The Netherlands; 4 Michigan Neuroscience Institute, University of Michigan, Michigan, USA; 5 Department of Medicine, Medical College East Africa, The Aga Khan University, Nairobi, Kenya; 6 Department of Biostatistics, University of Michigan, Ann Arbor, MI USA; 7 Department of Population Health, Aga Khan University, Srijan Sen, Nairobi, Kenya; 8 Department of Community Health and Epidemiology, Dalhousie University, Halifax, Canada; 9 Computing and Data Innovation Office, Aga Khan University, Nairobi, Kenya; 10 Center for Global Health Equity, University of Michigan, Ann Arbor, MI USA; 11 Department of Learning Health Sciences, University of Michigan Medical School, Ann Arbor, MI USA; 12 Department of Sociology, University of Nairobi, Nairobi, Kenya; 13 Institute for Human Development, Aga Khan University, Nairobi, Kenya; 14 Neurosciences Unit, Kenya Medical Research Institute?Wellcome TrustResearch Programme, Kilifi, Kenya

**Correction: BMC Research Notes (2023) 16:276**

10.1186/s13104-023-06498-6

Following the publication of the original article, we were informed that co-author Elena Frank’s first and last names were accidentally interchanged.

The correct name is shown in the author list of this Correction. The original article has been revised.

